# MicroRNA profiling in bovine serum according to the stage of *Mycobacterium avium* subsp. *paratuberculosis* infection

**DOI:** 10.1371/journal.pone.0259539

**Published:** 2021-11-04

**Authors:** Sung-Woon Choi, Suji Kim, Hong-Tae Park, Hyun-Eui Park, Jeong-Soo Choi, Han Sang Yoo

**Affiliations:** 1 Department of Infectious Disease, College of Veterinary Medicine, Seoul National University, Seoul, Republic of Korea; 2 BK21 FOUR Future Veterinary Medicine Leading Education and Research Center, College of Veterinary Medicine, Seoul National University, Seoul, Republic of Korea; 3 Research Institute for Veterinary Science, College of Veterinary Medicine, Seoul National University, Seoul, Republic of Korea; 4 Department of Microbiology, Research Institute of Life Science, College of Medicine, Gyeongsang National University, Jinju, Republic of Korea; 5 Bacterial Disease Division, Animal and Plant Quarantine Agency, Gimcheon-si, Republic of Korea; Cornell University, UNITED STATES

## Abstract

*Mycobacterium avium* subsp. *paratuberculosis* (MAP) is the causative agent of Johne’s disease (JD), and it causes diarrhea and weakness in cattle. During a long subclinical stage, infected animals without clinical signs shed pathogens through feces. For this reason, the diagnosis of JD during the subclinical stage is very important. Circulating miRNAs are attracting attention as useful biomarkers in various veterinary diseases because of their expression changes depending on the state of the disease. Based on current knowledge, circulating miRNAs extracted from bovine serum were used to develop a diagnostic tool for JD. In this study, the animals were divided into 4 groups according to fecal shedding, the presence of antibodies, and clinical signs. Gene expression was analyzed by performing miRNA sequencing for each group, and it was identified that the miRNA expression changed more as the MAP infection progressed. The eight miRNAs that were differentially expressed in all infected groups were selected as biomarker candidates based on their significant differences compared to the control group. These biomarker candidates were validated by qRT-PCR. Considering the sequencing data, two upregulated miRNAs and two downregulated miRNAs showed the same trend in the validation results. Network analysis was also conducted and the results showed that mRNAs (*IL-10*, *TGF-β1*) associated with regulatory T cells were predicted to be activated in the subclinical stage. Taken together, our data suggest that two miRNAs (bta-miR-374b, bta-miR-2887) may play major roles in the immune response to MAP infection during the subclinical stage.

## Introduction

*Mycobacterium avium* subsp. *paratuberculosis* (MAP) is the causative agent of paratuberculosis (PTB) or Johne’s disease (JD), which is a chronic infectious disease characterized by persistent diarrhea and debilitation in cattle [1–3]. Before clinical symptoms develop, it causes a great loss to the livestock industry due to a decline in milk production and the reproduction rate [4]. MAP, which is generally known to cause disease in ruminants, is also associated with various autoimmune diseases in humans [5]. Crohn’s disease (CD), a chronic inflammatory bowel disease, continues to be debated due to its similarity to JD [6]. Given this, MAP is considered to be important as a potential zoonotic agent.

According to the clinical signs and MAP excretion, there are four stages: silent, subclinical, clinical and advanced [1]. During a long subclinical stage, infected animals excrete small amounts of MAP in their feces without clinical signs. Once contaminated with MAP, most of their cohabitating livestock are infected, making the disease difficult to eradicate [3]. Therefore, it is important to diagnose JD during the subclinical stage, but current diagnostic methods such as serum ELISA and fecal PCR tests are not accurate for diagnosis [7–9]. As a result, a reliable early diagnostic tool for JD is essential. Recently, attempts have been made to overcome the difficulty of diagnosing MAP in the subclinical stage. Differentially expressed serum proteins were identified by proteomic profiling and ELISA was performed to discover a potential novel biomarker called A2M [10]. There was also a study using the NanoString nCounter technology that suggested a combination of four miRNAs that are distinguished from the negative control group as biomarkers according to the severity of JD [11].

MicroRNAs are noncoding RNA molecules consisting of approximately 22 nucleotides that regulate mRNA expression and have roles in posttranscriptional control [12,13]. Since these roles are closely linked to the causes of diseases, they are now being studied extensively in human and veterinary medicine [14,15]. Circulating miRNAs in serum, plasma or other body fluids are highly conserved and protected from the endogenous activity of RNases [16]. Additionally, the use of a combination of miRNAs is a noninvasive method [17] and could be used to monitor disease progression, as circulating miRNAs vary in their expression levels depending on the status of the disease [18–20]. These features make miRNAs potential diagnostic biomarkers for various types of diseases [21–24] and have recently attracted attention as a potential solution for MAP diagnosis. In this regard, attempts have been undertaken to develop diagnostic biomarkers by extracting microRNA from the serum, whole blood, and feces of JD-infected cattle [18,25,26]. A previous study on miRNA extraction from serum suggested biomarkers by comparing the IFN-gamma positive group, which were experimentally infected MAP with the negative group [25]. Another previous study on miRNAs extracted from whole blood and feces compared one MAP-infected group with a negative control group [18,26]. Therefore, this study suggested the early diagnostic biomarkers of JD according to the infection stage by the results of ELISA, PCR, and clinical signs.

MAP is an intracellular pathogen and it resides in host macrophages. In MAP-infected macrophages, apoptosis is suppressed, thus limiting bacterial destruction and enhancing their survival [27]. In response to MAP infection, the Th1 immune response begins with proinflammatory cytokines. As it progresses to the clinical stage, the Th1 immune response decreases, and the Th2 immune response becomes predominant. At this point, the activity of anti-inflammatory cytokines appears [28,29]. The exact mechanism of this immune shift is unknown, but the speculative possibility is a population of regulatory T cells (Tregs). Tregs suppress immune responses, and the interferon-gamma reduction that occurs during subclinical to clinical progression is caused by Tregs limiting the response of effector T cells [27,30]. To develop an early diagnosis of JD, it is necessary to identify the immune response that changes according to the infection status and related factors.

In this study, we purified miRNA from bovine serum and conducted miRNA sequencing to analyze differentially expressed miRNAs of the MAP-infected group compared to the negative controls. According to the JD stages, we identified the changes in the profile of the miRNAs and the immune response that occurs when they are infected through the network between miRNAs and related genes. We confirmed the potential of miRNAs as biomarkers for the early diagnosis of JD.

## Materials and methods

### Experimental animals and serum samples

Cattle were screened from several farms in Chungcheongnam and Gangwon provinces of South Korea from 2013 to 2019 for the diagnosis of JD. A total of 298 serum samples were obtained during this period [31], and the samples selected according to the previously described criteria were used in this experiment, including miRNA analysis and validation. First, for the small RNA sequencing of miRNA in serum samples, we divided the animals into four groups and selected experimental samples based on fecal MAP PCR, serum ELISA, and clinical signs. Group A (n = 4) was PCR- and ELISA-positive and showed clinical signs; Group B (n = 8) was also PCR- and ELISA-positive but showed no clinical signs; Group C (n = 8) was only PCR-positive; and Group D (n = 8) was used as a control in this study. These samples were pooled for sequencing analysis. Specifically, the serum of the cows in Group A was collected before death due to severe clinical symptoms, and Group C was defined as an early subclinical infection stage with positive fecal shedding. Next, experiments to validate the miRNA-sequencing results were grouped according to the above criteria, but the number of samples was different. The number of samples in Group A (n = 4) was the same, and Groups B (n = 11), C (n = 28), and D (n = 46) added samples for validation by real-time PCR, including those used for sequencing. The mGITC/SC method [32] was used for DNA extraction from animal feces, and real-time PCR was conducted to detect a combination of IS900 and IS*MAP*02 amplification [33]. Next, the results of the serum ELISA were obtained by using a commercial ELISA kit (IDEXX Laboratories, Inc., USA). The naturally infected cows with symptoms showed persistent diarrhea and weight loss, which eventually led to death. Following the study, the cattle in group A were led to death, while the cattle in the other groups were managed in isolation from their respective farms. This study was conducted in strict accordance with the guidelines of the Institutional Animal Use and Care Committee of the National Institute of Animal Science. The protocol was approved by the Institutional Animal Use and Care Committee of the National Institute of Animal Science (2013–046) and the Animal Ethics Committee of Seoul National University (SNU-200525-4).

### RNA extraction

Total RNA was extracted from bovine serum using the miRNeasy Serum/Plasma Kit (Qiagen, Germany) according to the manufacturer’s instructions with a few modifications. Two hundred microliters of individual bovine serum was used and finally eluted in 14 μl RNase-free water. The total RNA was then measured using a Nanodrop ND-1000 instrument (Thermo Fisher Scientific, MA, USA).

### Small RNA sequencing

The libraries were prepared with the NEXTflex Small RNA-Seq Kit for 50 bp single-end sequencing. (Bio Scientific Corp.). More precisely, small RNA molecules were isolated from 1 μg of total RNA through ligature of the adapter, and the isolated small RNAs were synthesized into single-stranded cDNAs through reverse transcription priming. Double-stranded cDNA was prepared by PCR and applied as a template for the synthesis of the second strand, and fragments of approximately 150 bp were isolated for sequencing using an electrophoresis gel for size selection. The quality of these cDNA libraries was assessed using the Agilent 2100 BioAnalyzer (Agilent, CA, USA) followed by quantification with the KAPA library quantification kit (Kapa Biosystems, MA, USA), in accordance with the manufacturer’s protocol. After cluster amplification of denatured templates, sequencing was performed with paired-end sequencing (150 bp) using an Illumina NovaSeq 6000 (Illumina, CA, USA).

### miRNA-seq data analysis

Low-quality reads were trimmed or filtered for bases with a quality score of less than 20 and a read length of less than 17 bp, which was conducted with the help of the Cutadapt tool [34]. Filtered reads were mapped to the reference genome of the relevant species using the aligner Bowtie [35], and then variant calling in the miRNA seed region was performed. Variant calling was conducted in the miRNA seed region using GATK to look for variations [36], and the maximum depth threshold in the region was set to 1,000 using the dcov option. The expression level of miRNA was assessed with mirdeep2 [37] using the species’ gene annotation database, as well as hairpin and mature miRNA sequence information obtained from miRBase [38], and all of the parameters were set to default values. For the analysis of differentially expressed miRNAs (DE-miRNAs), miRNA level count data were produced using mirdep2, and DE-miRNAs were identified using the R package TCC [39] based on the computed read count data. Normalization factors were computed using the iterative DEGES/edgeR method, and the Q value was computed on the basis of the p value using the R package with default parameter settings. The DE miRNAs were identified using a q-value cutoff less than 0.05 to correct for multiple-testing errors [40]. The raw miRNA sequencing data were submitted to GEO with accession number GSE166230. Hierarchical cluster analysis (HCA) of the DE-miRNAs per group was performed by using Multi-Experimental Viewer (MeV) software version 4.9.0. and processed based on the Euclidean distance metric and average linkage clustering.

### Target prediction and network analysis

We used TargetScanHuman 7.2 (http://www.targetscan.org/vert 72/) to predict the gene targets of the selected DE-miRNAs, with the threshold set at a cumulative weighted context++ score ≤ −0.2 [41]. The PANTHER Classification System (http://pantherdb.org/) [42] was used to conduct gene ontology (GO) analyses, and the DAVID bioinformatics online tool (https://david.ncifcrf.gov/) [43] was used to perform Kyoto Encyclopedia of Genes and Genomes (KEGG) pathway analyses on the genes predicted by up- or downregulated selective miRNAs.

Network analysis was conducted by Ingenuity Pathway Analysis (IPA; Qiagen Inc., https://www.qiagenbioinformatics.com/products/ingenuitypathway-analysis) [44] software to confirm the molecules and expression patterns related to the selected miRNAs. As bovine mature miRNA has not been annotated to the IPA database, it was necessary to convert it to human mature miRNA. The sequences identified as mature miRNAs of different species were derived from the same precursor miRNAs of specific species, which were then converted into human miRNAs. Through this process, we performed a network analysis using the converted miRNAs. From the selected miRNAs, IPA showed interactions by predicting and connecting the target molecules.

### Quantitative real-time PCR for the validation of miRNA sequencing data

For first-strand cDNA synthesis, we used the miRCURY LNA RT kit (Qiagen, Germany). The total reaction volume was 10 μl, and UniSp6 RNA spike-in was included in all reactions to evaluate the cDNA synthesis. We then diluted the synthesized cDNA to 1:20 in RNase-free water. Next, qRT-PCR was performed using the miRCURY LNA SYBR^®^ Green PCR Kit (Qiagen) and a customized miRCURY LNA miRNA PCR assay (Qiagen) targeting the miRNAs detected by miRNA sequencing. The predesigned UniSp6 assay was used as an internal PCR control, and each reaction was performed on a Rotor-Gene Q real-time PCR cycler (Qiagen) starting at 95°C for 2 min, followed by 40 cycles of 95°C for 10 s and 56°C at 60 s.

Statistical analyses were performed using GraphPad Prism software version 7.00 (GraphPad Software, USA), and the results are presented as the mean with SEM of independent experiments (n = 4 for Group A, n = 11 for Group B, n = 28 for Group C, n = 46 for Group D). The relative changes in gene expression were calculated using the 2^−ΔΔCt^ method with UniSp6 as a reference gene. The fold changes are represented by the mean ratio of gene expression in Groups A, B and C compared to Group D. qRT-PCR data was analyzed using non-parametric Kruskal-Wallis test with Dunn’s post-hoc test. Statistical significance was considered as a P-value of less than 0.05.

## Results

### Identification of the differentially expressed miRNAs

**Fig 1:**
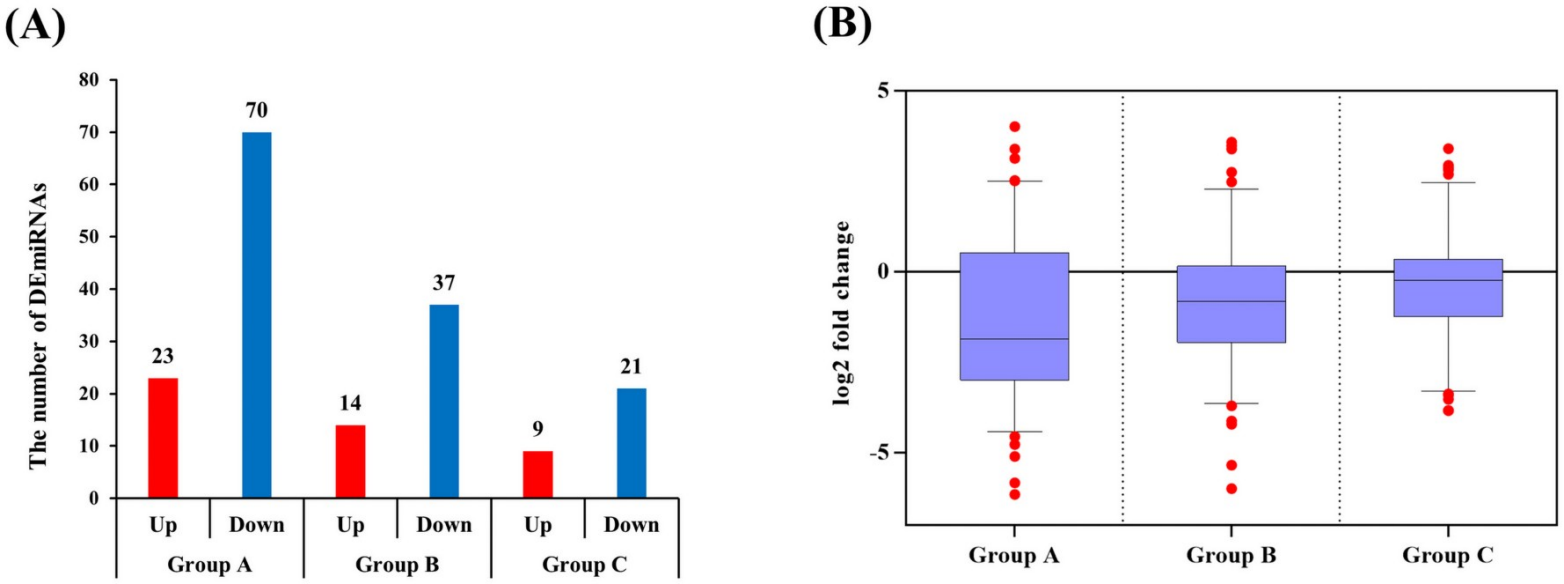
Results of the miRNA-sequencing analysis. (A) The number of DE-miRNAs in bovine serum infected with *Mycobacterium avium* subsp. *paratuberculosis*. compared to the control serum. (B) The fold changes of DE-miRNAs in the diseased groups are depicted using box and whisker (5–95 percentile) plots.

**Fig 2:**
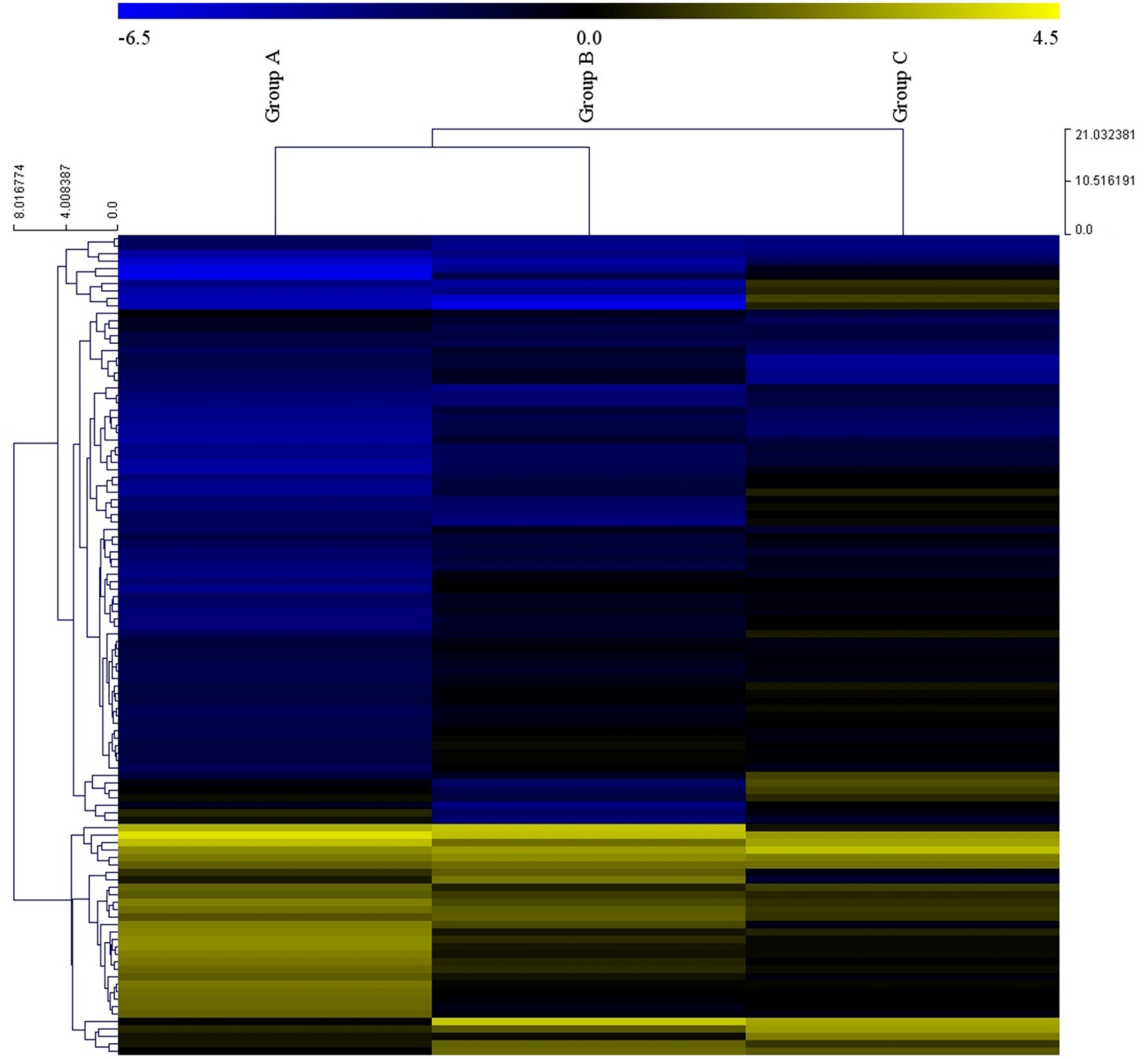
Clustering analysis of MAP-infected groups compared with the noninfected group. Each row in the heat map represents miRNAs, and each column represents samples. Yellow indicates increased expression, and blue indicates decreased expression.

Sequencing was performed and then we compared the control group (Group D) to each case group (Groups A, B and C) to identify the DE-miRNAs. In this study, the log2-fold change value was used to determine whether the expression was upregulated or downregulated compared to the control group. A total of 110 common DE-miRNAs were identified in all disease groups, and we decided that the miRNAs with | log2 fold change | ≥ 1.5 were significant. Ninety-three miRNAs in Group A, fifty-one miRNAs in Group B and thirty miRNAs in Group C were differentially expressed, and there were more miRNAs with decreased expression levels than increased expression levels in all groups (Fig 1A). Moreover, the fold change of DE-miRNAs compared to the controls showed greater changes in miRNA expression as they progressed to the clinical stage (Fig 1B). The heatmap of the DE-miRNAs in each group showed that Group B tended to be more similar to Group A than Group C (Fig 2). This means that cattle with positive fecal PCR and serum ELISA were closer to the clinical stage of JD.

Thereafter, we selected eight miRNAs as biomarker candidates, which were differentially expressed as significantly increased or decreased in all groups relative to the control group (Table 1). These selected miRNAs expressed more than 2-fold changes in all groups. Three miRNAs were upregulated (bta-miR-363, bta-miR-374b, bta-miR-2887), and five miRNAs were downregulated (bta-miR-147, bta-miR-196a, bta-miR-346, bta-miR-655, bta-miR-2426).

**Table 1:** Selective miRNA sequence and fold change in the expression of miRNA compared with the control group.

Target miRNA	Human ortholog	miRNA sequence	Log2 fold change^a^		
			Group A	Group B	Group C
**bta-miR-363**	hsa-miR-363-5p	ATTGCACGGTATCCATCTGCG	2.49	2.75	3.41
**bta-miR-374b**	hsa-miR-374b-5p	ATATAATACAACCTGCTAAGTG	4.02	3.39	2.70
**bta-miR-2887**	-	CGGGACCGGGGTCCGGTGCG	2.15	2.49	2.25
**bta-miR-147**	hsa-miR-147b	GTGTGCGGAAATGCTTCTGCTA	-2.45	-3.32	-3.25
**bta-miR-196a**	hsa-miR-196a-5p	TAGGTAGTTTCATGTTGTTGGG	-3.45	-2.08	-2.43
**bta-miR-346**	hsa-miR-346	TGTCTGCCCGCATGCCTGCCTCT	-5.10	-4.13	-2.23
**bta-miR-655**	hsa-miR-655-5p	ATAATACATGGTTAACCTCTCT	-2.54	-3.58	-3.41
**bta-miR-2426**	-	AAGGAAGTGGCTTGGGGAAAG	-4.16	-3.20	-2.98

^a^Log2 fold change in the infection groups compared to the control group.

### Functional classification of the miRNA targets and ingenuity pathway analysis

**Fig 3:**
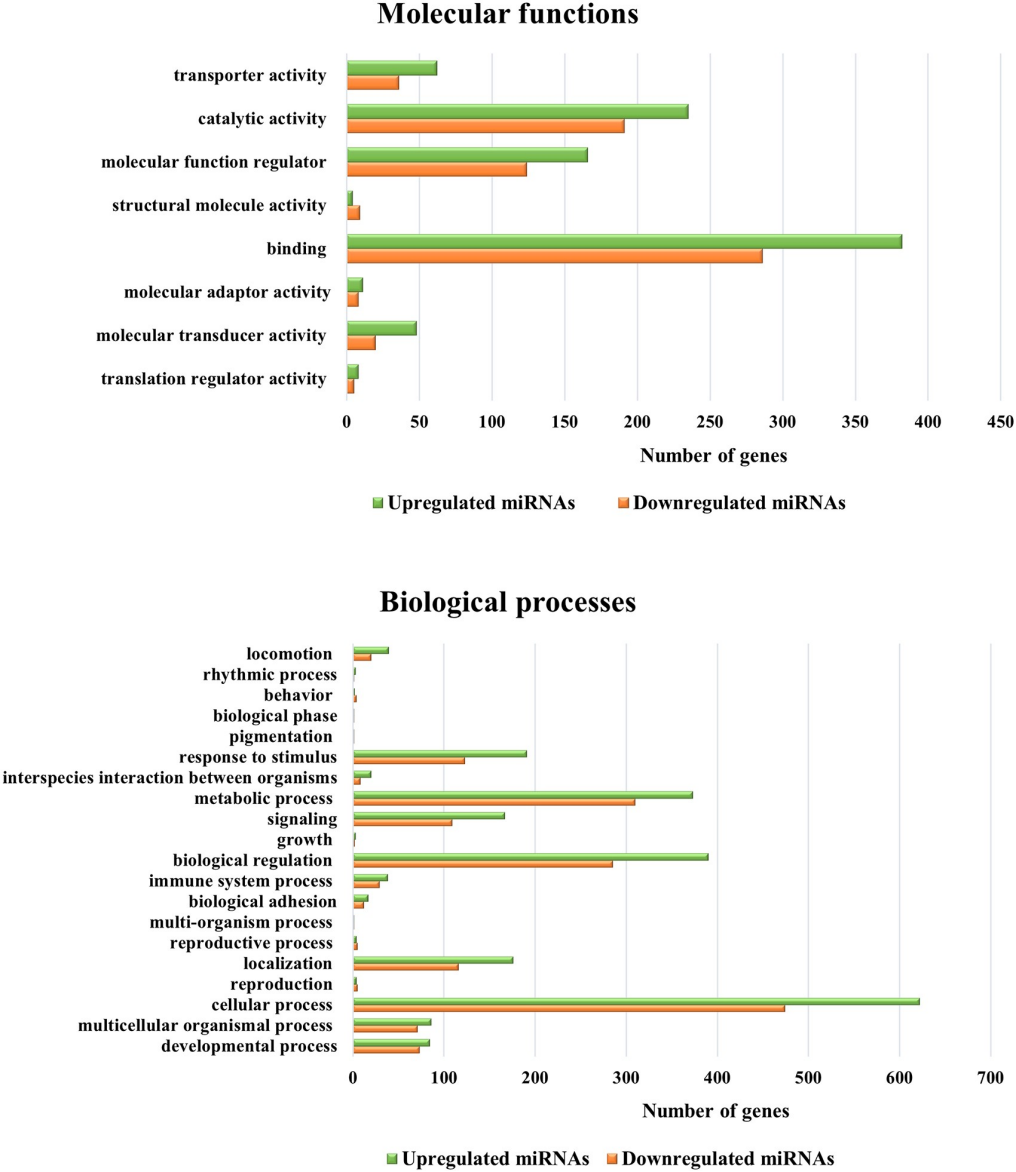
GO analysis of gene targets of selective miRNAs. These gene targets were classified into molecular functions (A) and biological processes (B) using the gene ontology-based online tool, the PANTHER Classification System.

**Fig 4:**
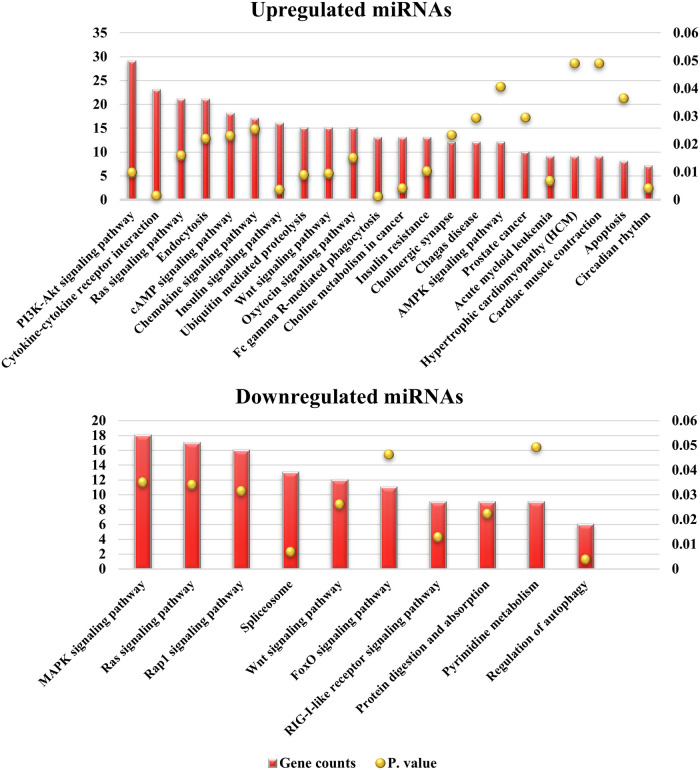
KEGG analysis of gene targets of the selected miRNAs. List of genes predicted by upregulated (A) and downregulated (B) selective miRNAs showed various pathways involvement. Only P values less than 0.05 were included in the analyses.

The predicted targets of the up- and downregulated selective miRNAs were 1240 and 1022 genes, respectively (S1 Table). The GO analysis of the target genes indicated that 8 and 20 GO terms were enriched in molecular function and biological processes, respectively. The target genes were shown to be associated with binding, molecular function regulator, catalytic activity and transporter activity in molecular function (Fig 3A). On the other hand, the biological process terms included cellular process, biological regulation, metabolic process, response to stimulus and immune system process (Fig 3B). Next, KEGG analysis of the target genes of up- and downregulated miRNAs revealed a number of pathways. The upregulated miRNAs mostly targeted signaling pathways such as PI3K-Akt, Ras, cAMP, chemokine and cytokine-cytokine receptor interaction and endocytosis, while the downregulated miRNAs mostly targeted signaling pathways such as MAPK, Ras, Rap1, Wnt and pathways in cancer (Fig 4A and 4B).

**Fig 5:**
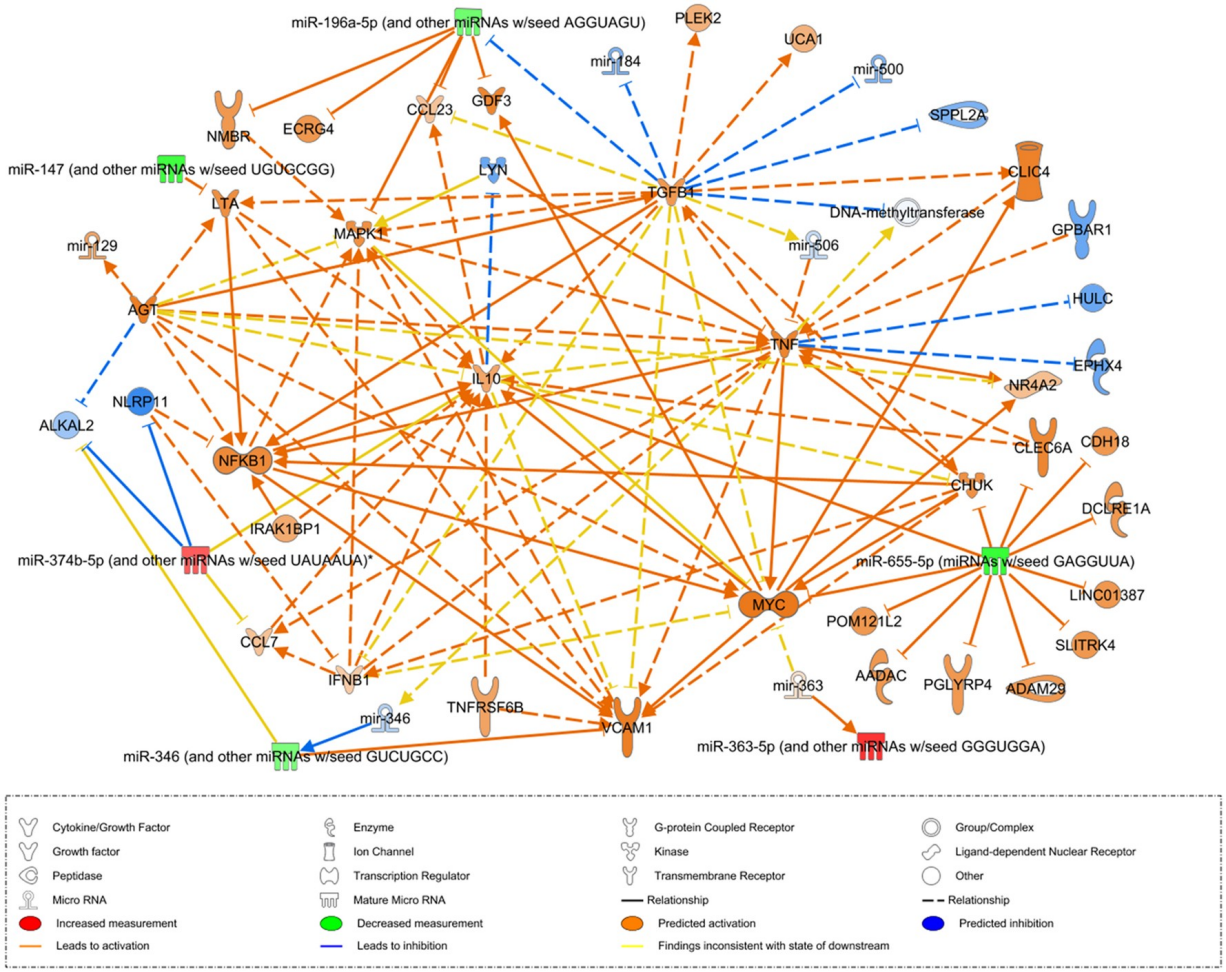
Common network of DE-miRNAs in early subclinical *Mycobacterium avium* subspecies. *paratuberculosis* infected group. Upregulation (red) or downregulation (green) of the miRNAs and predicted increase (orange) or decrease (blue) in the molecules.

As previously mentioned in the Materials and methods, the bovine miRNAs were converted into human orthologues to proceed with the IPA (S2 Table). The significantly expressed genes were selected by IPA based on the | log2 fold change | ≥ 1.5 after the bovine gene ID was converted to the human gene ID. Eighty-four DEGs were expressed in Groups A, B and C in comparison to Group D. We attempted to identify the molecules associated with the miRNA through IPA software by mapping eight miRNAs selected from the sequencing analysis, but two bovine miRNAs (bta-miR-2426, bta-miR-2887) were not mapped because they lacked human miRNA homologs. We performed the network analysis with the six converted miRNAs in the early subclinical infection stage (Group C). A total of 51 molecules, including the six selected miRNAs, were linked, and these targets were predicted through their changes in miRNA expression (Fig 5). This common network of DEGs is involved in many biological processes, including the inflammatory response, immune response, signal transduction and cell-cell signaling. *TGF-β1* and *IL-10*, which are at the center of the common network, are activated at an early subclinical stage and have been identified as implicated in immune responses.

### Validation of selective differentially expressed miRNAs

**Fig 6:**
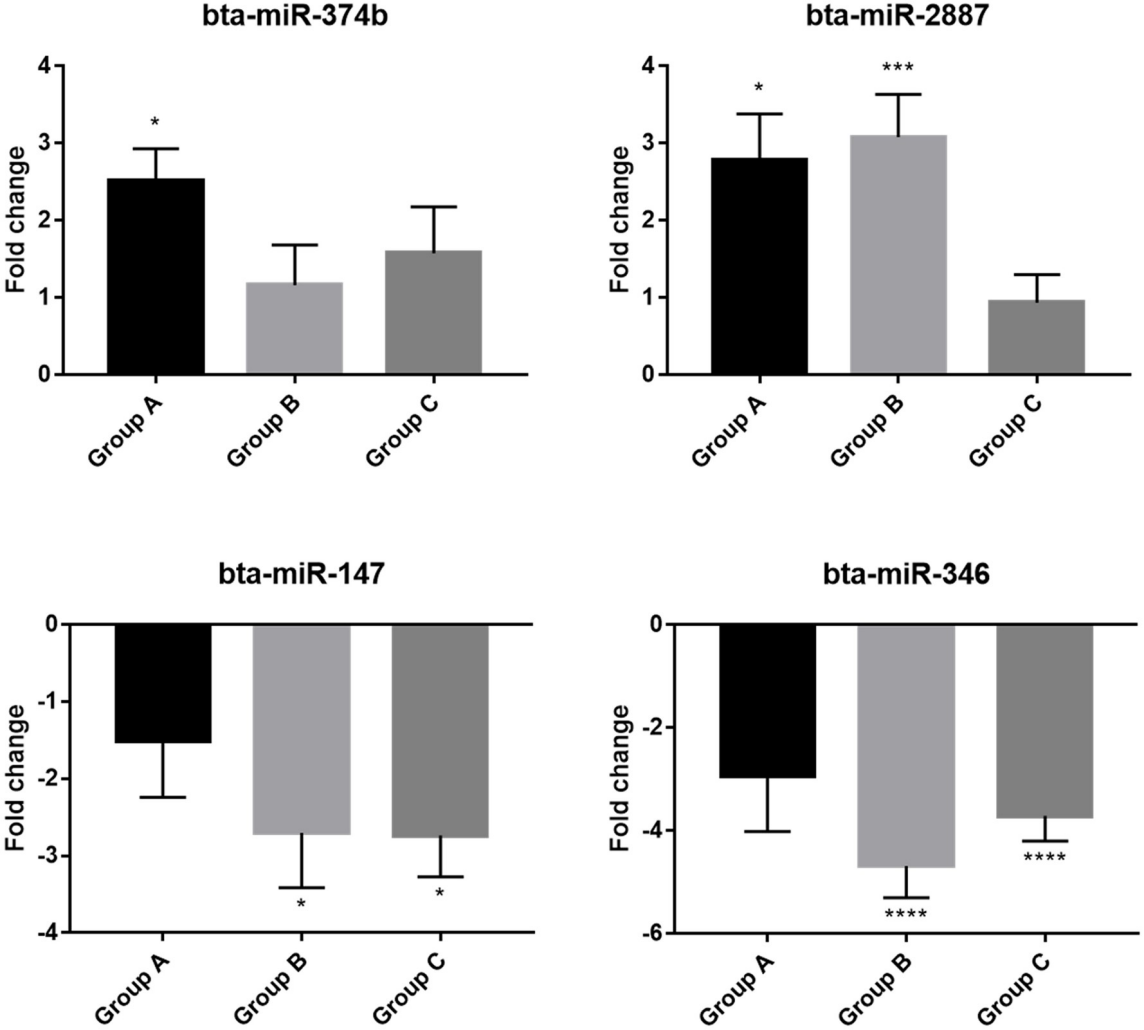
Gene expression profiling of selected miRNAs by qRT-PCR. qRT-PCR was used for validation of the miRNA sequencing results. Each bar represents the mean ± SEM from independent experiments in individual cows. Statistical significance was determined using the non-parametric Kruskal-Wallis test with Dunn’s post-hoc test. (p-value, * < 0.05; ** < 0.01; *** < 0.0005; **** < 0.0001).

Eight selected miRNAs were validated by using qRT-PCR. Including the serum samples used for sequencing, validation was conducted by adding serum samples based on criteria suitable for each group Among them, 4 DE-miRNAs showed significant differences in expression compared to the control group. (Fig 6). Two upregulated miRNAs (bta-miR-374b, bta-miR-2887) and two downregulated miRNAs (bta-miR-147, bta-miR-346) showed a consistent pattern with the sequencing experiments (Table 1). The validation results revealed that the expression levels of bta-miR-374b and bta-miR-2887 showed upregulation significantly in Group A, which were clinical stages of JD infection. In contrast, two downregulated bta-miR-147 and bta-miR-346 were significantly found in Groups B and C which showed no clinical signs.

## Discussion

Johne’s disease (JD) is a chronic disease that infects livestock, and then they intermittently excrete the bacteria in their feces for 2 to 5 years without exhibiting any obvious symptoms [2]. Early diagnosis is important because of this characteristic, but the present diagnostic techniques are difficult to use due to the intermittent nature of MAP fecal shedding and the poor sensitivity of serological testing [9]. Therefore, it is necessary to develop a diagnostic tool for detecting MAP in the subclinical stage.

In this study, we conducted experiments by defining groups based on the clinical symptoms and the results of fecal PCR and serum ELISA, which are currently used for the diagnosis of JD. Then, we performed miRNA sequencing with serum samples from each group and confirmed the DE-miRNAs compared to the control group. The group with the clinical symptoms (Group A) had the most DE-miRNAs, and the change pattern was also larger than the other groups when comparing the expression levels of the common DE-miRNAs based on | log2 fold change | ≥ 1.5. Among these DE-miRNAs, we selected those that exhibited up- or downregulation in all disease groups as potential biomarker candidates. To select a miRNA biomarker for the early diagnosis of disease, it is necessary to show a constant expression level during different stages of the disease. Furthermore, because numerous diseases may have similar miRNA expression patterns, it is important to select miRNA biomarkers that may distinguish them from other diseases [45].

Through the sequencing results, we initially identified three upregulated miRNAs and five downregulated miRNAs as potential biomarkers. All of these candidate miRNAs have been reported to be associated with bacterial or viral infection in cattle [46–52]. Bta-miR-363, one of these candidate miRNAs, was identified in the serum of JD-infected cattle in a previous study, and its expression was shown to be increased when compared to the negative control group [11]. Moreover, bta-miR-363 was also upregulated in milk-isolated monocytes infected with *Streptococcus uberis* [46]. Although the exact mechanism of action needs to be studied, it seems that bta-miR-363 is upregulated during infection with Gram-positive bacteria. Likewise, bta-miR-374b and bta-miR-2887, which were upregulated in our study, were also upregulated by *Streptococcus agalactiae* and *Staphylococcus aureus* infection of monocyte-derived macrophages and mammary gland tissue [47,48]. Three downregulated miRNAs (bta-miR-196a, bta-miR-147, bta-miR-655) also changed their levels of expression during *S*. *aureus* infection; bta-miR-196a and bta-miR-655 were downregulated, whereas bta-147 was upregulated [48,51]. Specifically, downregulated bta-miR-196a and bta-miR-655 were isolated from mammary gland tissue, whereas upregulated bta-miR-147 was isolated from milk, implying that the regulatory shift was caused by a difference in the miRNA source. In addition, the human homolog of bta-miR-196a was overexpressed in the inflammatory intestinal epithelium of patients with Crohn’s disease associated with MAP [53]. A human homolog of bta-miR-346 has been identified as a potential biomarker that is upregulated during tuberculosis and *M*. *avium* complex (MAC) pulmonary disease caused by *Mycobacterium* species [54,55]. Ruminant-specific bta-miR-2426 is a novel miRNA discovered during bovine herpesvirus 1 infection, and there have been no reports of further detection due to other infections [52].

Next, target genes of upregulated and downregulated miRNAs were predicted using TargetScan. Other target prediction tools, such as Diana Tools and mirPath, are accessible, but only TargetScan was able to predict bovine target genes. GO analysis showed that these miRNAs were mainly involved in cell processes, metabolic processes, biological regulation, and responses to stimuli. This result was also related to reported research that showed that MAP survives in infected macrophages by involving various metabolic processes, such as lipid metabolism [56].

KEGG analysis revealed that the target genes are primarily associated with the signaling pathway and they are commonly enriched in the Ras and Wnt signaling pathways. Ras controls a variety of cellular processes, such as cell survival, proliferation, and differentiation, all of which are necessary for a proper immune response [57]. It also triggers the Raf/Mek/Erk, PI3K, and RalGDS signaling pathways. The ERK (MAPK) pathway may have a role in inducing proinflammatory responses in MAP-infected cattle, and the PI3K pathway is involved in macrophage adherence and motility by suppressing proinflammatory cytokines [58,59]. The Wnt signaling pathway is involved in pathological processes and it regulates effector T cell development, regulatory T cell activation, and dendritic cell maturation in the immune system [60]. The *Wnt* gene appears to be involved in the development of granulomas in MAP-infected cattle by recruiting immune cells to the inflammatory site [61].

Taken together, these findings indicated that the dysregulated miRNAs are involved in pathways controlling immune and inflammatory responses during MAP infection. After identifying the function of the DE-miRNAs, network analysis was performed using IPA to confirm the interaction between the miRNAs and other genes. Except for the two ruminant-specific miRNAs (bta-miR-2887 and bta-miR-2426), the human homologs of the six bovine miRNAs were mapped to IPA. Many molecules were linked, directly or indirectly, with cytokine factors and transcription regulators such as *TNF*, *IL10*, *TGF-β1*, and *NFKB1* served as the center of the network. TNF is a key proinflammatory cytokine in the early stages of MAP infection, inducing a Th1 immune response by limiting intracellular growth [62]. *MYC* stimulates the production of this proinflammatory cytokine, which aids in the inhibition of MAP intracellular growth [63]. TNF is also a key mediator in granuloma formation, and TNF-induced *VCAM-1* is involved in lymphocyte adherence to inflamed endothelium [64]. This network activates *TGF-β1* and *IL-10*, which are produced by Tregs that develop as a result of chronic MAP antigen stimulation [30]. Furthermore, *LYN* expression was decreased among the key genes related to the immune response, while the expression levels of *CCL23*, *CCL7*, *TNFRSF6B*, *CHUK* were increased and previous studies have identified these genes as implicated in the immune response to mycobacterial infection [56,65–67].

In JD-infected cattle, a cytokine imbalance in which immunoregulatory cytokines such as TGF-β1 and IL-10 take priority over IFN-γ production is thought to mediate the transition from the subclinical to clinical stage [27]. That is, the Th-1 cell-mediated immune response gradually decreases in the subclinical stage, making it difficult to suppress intracellular infection of MAP, and the disease progresses to the clinical stage. Additionally, NFKB1 promotes the production of anti-inflammatory IL-10, which suppresses inflammation, and MAPK also contributes to IL-10 production, allowing MAP to survive intracellularly [68]. Overall, the network reveals that both Th-1-related molecules that develop early in MAP infection and Th-2 and Treg-related molecules that are prominent in later infection are activated. This indicates that the network meets the criteria for early diagnostic biomarkers that should have a constant level of expression at all stages by exhibiting the overall immune response in the JD infection group.

Finally, a validation experiment was conducted to identify the expression of miRNAs in all infected groups. Two upregulated miRNAs (bta-miR-374b, bta-miR-2887) and two downregulated miRNAs (bta-miR-147, bta-miR-346) were differentially expressed with significant changes. Among them, bta-miR-147 and bta-miR-346 were significantly found in Groups B and C, which were subclinical stages before clinical signs appeared. On the other hand, bta-miR-374b and bta-miR-2887 were shown to be significantly different in Group A compared to the control group (P < 0.05). These two upregulated miRNAs, which were significantly expressed in Group A with severe clinical symptoms, showed the same expression pattern in Group B and C, suggesting that they could be used as potential miRNA biomarkers in the early stage of JD.

As such, our study profiled miRNAs extracted from serum samples at different stages of MAP infection and identified the immune response to MAP infection. Selective miRNAs commonly expressed at different stages of MAP infection have been identified, and the use of these microRNA biomarkers is expected to play an essential role in future JD management and prevention.

## Supporting information

S1 Table Target genes of the up- and downregulated selective miRNAs. (XLSX)

S2 Table Converted human miRNAs mapped to IPA. (DOCX)
