# Supplementary material for: MicroRNA profiling in bovine serum according to the stage of Mycobacterium avium subsp. paratuberculosis infection
Source: PLoS One. 2021 Nov 4;16(11):e0259539. doi: 10.1371/journal.pone.0259539 (PMC8568169; doi:10.1371/journal.pone.0259539)
Supplement: S2 Table — (DOCX) [file pone.0259539.s002.docx]

**S2 Table. Converted human miRNAs mapped to IPA.**

| Bovine mature ID | Bovine target sequence | Human converted ID | Group A | Group B | Group C |
| --- | --- | --- | --- | --- | --- |
|  |  |  | Log2 fold change | Log2 fold change | Log2 fold change |
| bta-let-7b | TGAGGTAGTAGGTTGTGTGGTT | hsa-let-7b-5p | 2.258 | 0.367 | 0.173 |
| bta-let-7c | TGAGGTAGTAGGTTGTATGGTT | hsa-let-7c-5p | 2.109 | 0.129 | 0.149 |
| bta-let-7g | TGAGGTAGTAGTTTGTACAGTT | hsa-let-7g-5p | 2.522 | 0.805 | 0.174 |
| bta-let-7i | TGAGGTAGTAGTTTGTGCTGTT | hsa-let-7i-5p | 1.905 | -0.188 | -0.028 |
| bta-miR-1 | TGGAATGTAAAGAAGTATGTAT | hsa-miR-1-3p | -4.779 | -5.342 | 1.196 |
| bta-miR-100 | AACCCGTAGATCCGAACTTGTG | hsa-miR-100-5p | -1.767 | -0.184 | 0.166 |
| bta-miR-10b | TACCCTGTAGAACCGAATTTGTG | hsa-miR-10b-5p | -1.731 | -0.343 | 0.379 |
| bta-miR-122 | TGGAGTGTGACAATGGTGTTTG | hsa-miR-122-5p | -2.961 | -1.225 | -0.031 |
| bta-miR-1249 | ACGCCCTTCCCCCCCTTCTTCA | hsa-miR-1249-3p | -3.528 | -2.153 | -1.413 |
| bta-miR-124a, bta-miR-124b | TAAGGCACGCGGTGAATGCCAAG | hsa-miR-124-3p | -1.852 | -1.325 | -3.832 |
| bta-miR-125a | TCCCTGAGACCCTTTAACCTGTG | hsa-miR-125a-5p | -3.152 | -1.747 | -0.055 |
| bta-miR-129, bta-miR-129-5p | CTTTTTGCGGTCTGGGCTTGCT | hsa-miR-129-5p | -3.131 | -2.811 | -1.722 |
| bta-miR-1306 | CCACCTCCCCTGCAAACGTCC | hsa-miR-1306-5p | -3.192 | -1.762 | -0.681 |
| bta-miR-130a | CAGTGCAATGTTAAAAGGGCAT | hsa-miR-130a-3p | -2.252 | -1.055 | -2.448 |
| bta-miR-133a | TTTGGTCCCCTTCAACCAGCTG | hsa-miR-133a-3p | -3.421 | -4.211 | 0.881 |
| bta-miR-133b | TTTGGTCCCCTTCAACCAGCTA | hsa-miR-133b | -0.364 | -2.639 | 1.409 |
| bta-miR-139 | TCTACAGTGCACGTGTCTCCAGT | hsa-miR-139-5p | -1.584 | -0.313 | 0.046 |
| bta-miR-141 | TAACACTGTCTGGTAAAGATGG | hsa-miR-141-3p | -4.017 | -2.229 | -1.365 |
| bta-miR-143 | TGAGATGAAGCACTGTAGCTCG | hsa-miR-143-3p | -1.592 | 0.199 | -0.105 |
| bta-miR-144 | TACAGTATAGATGATGTACTAG | hsa-miR-144-5p | 1.885 | -0.656 | -0.025 |
| bta-miR-147 | GTGTGCGGAAATGCTTCTGCTA | hsa-miR-147b | -2.446 | -3.325 | -3.247 |
| bta-miR-16a | TAGCAGCACGTAAATATTGGTG | hsa-miR-16-1-3p | 2.511 | 0.430 | 0.143 |
| bta-miR-16b | TAGCAGCACGTAAATATTGGC | hsa-miR-16-5p | 1.980 | 0.038 | 0.045 |
| bta-miR-184 | TGGACGGAGAACTGATAAGGGT | hsa-miR-184 | -2.277 | -0.795 | -3.378 |
| bta-miR-185 | TGGAGAGAAAGGCAGTTCCTGA | hsa-miR-185-5p | 1.889 | 0.768 | 0.226 |
| bta-miR-186 | CAAAGAATTCTCCTTTTGGGCT | hsa-miR-186-5p | 2.113 | 0.669 | 0.042 |
| bta-miR-18a | TAAGGTGCATCTAGTGCAGATA | hsa-miR-18a-3p | 1.469 | 1.690 | 0.924 |
| bta-miR-192 | CTGACCTATGAATTGACAGCCAG | hsa-miR-192-5p | -2.732 | -0.771 | -0.188 |
| bta-miR-193a-5p | TGGGTCTTTGCGGGCGAGATGA | hsa-miR-193a-5p | -1.842 | -1.452 | -0.333 |
| bta-miR-194 | TGTAACAGCAACTCCATGTGGA | hsa-miR-194-5p | -2.090 | -0.585 | -0.600 |
| bta-miR-195 | TAGCAGCACAGAAATATTGGCA | hsa-miR-195-5p | -2.710 | -1.639 | -1.250 |
| bta-miR-196a | TAGGTAGTTTCATGTTGTTGGG | hsa-miR-196a-5p | -3.453 | -2.080 | -2.430 |
| bta-miR-197 | TTCACCACCTTCTCCACCCAGC | hsa-miR-197-3p | -2.809 | -1.391 | -0.712 |
| bta-miR-199a-5p | CCCAGTGTTCAGACTACCTGTT | hsa-miR-199a-5p | 0.741 | 1.582 | 2.810 |
| bta-miR-199b | CCCAGTGTTTAGACTATCTGTTC | hsa-miR-199b-3p | 0.261 | -1.590 | 0.707 |
| bta-miR-200a | TAACACTGTCTGGTAACGATGTT | hsa-miR-200a-3p | -1.899 | 0.135 | -0.494 |
| bta-miR-200b | TAATACTGCCTGGTAATGATG | hsa-miR-200b-5p | -1.580 | -0.447 | -0.426 |
| bta-miR-200c | TAATACTGCCGGGTAATGATGGA | hsa-miR-200c-3p | -2.323 | 0.018 | -0.760 |
| bta-miR-205 | TCCTTCATTCCACCGGAGTCTG | hsa-miR-205-5p | -2.137 | -0.050 | -0.368 |
| bta-miR-206 | TGGAATGTAAGGAAGTGTGTGG | hsa-miR-206 | -4.551 | -5.998 | 0.628 |
| bta-miR-20a | TAAAGTGCTTATAGTGCAGGTAG | hsa-miR-20a-5p | 1.600 | 1.026 | 0.680 |
| bta-miR-210 | ACTGTGCGTGTGACAGCGGCTGA | hsa-miR-210-3p | -2.235 | -0.891 | 0.491 |
| bta-miR-211 | TTCCCTTTGTCATCCTTTGCC | hsa-miR-211-3p | -1.809 | 0.081 | -0.214 |
| bta-miR-214 | ACAGCAGGCACAGACAGGCAGT | hsa-miR-214-3p | -1.971 | -0.977 | -0.397 |
| bta-miR-215 | ATGACCTATGAATTGACAGACA | hsa-miR-215-5p | -3.082 | -0.650 | -0.361 |
| bta-miR-218 | TTGTGCTTGATCTAACCATGTG | hsa-miR-218-5p | 3.136 | 3.489 | 0.338 |
| bta-miR-221 | AGCTACATTGTCTGCTGGGTTT | hsa-miR-221-3p | 1.655 | 0.340 | -0.376 |
| bta-miR-29b | TAGCACCATTTGAAATCAGTGTT | hsa-miR-29b-3p | -1.907 | -0.863 | -0.311 |
| bta-miR-30a-5p | TGTAAACATCCTCGACTGGAAGCT | hsa-miR-30a-5p | -2.112 | -0.727 | 0.209 |
| bta-miR-30f | TGTAAACACCCTACACTCTCAGCT | hsa-miR-30c-2-3p | -1.860 | -0.472 | 0.009 |
| bta-miR-323 | GCACATTACACGGTCGACCTCT | hsa-miR-323a-3p | -2.971 | -2.747 | 0.235 |
| bta-miR-328 | CTGGCCCTCTCTGCCCTTCCGT | hsa-miR-328-3p | -1.638 | -0.306 | -0.440 |
| bta-miR-335 | TCAAGAGCAATAACGAAAAATGT | hsa-miR-335-5p | -1.550 | -0.925 | 1.225 |
| bta-miR-33a | GTGCATTGTAGTTGCATTGCA | hsa-miR-33a-5p | -0.844 | -1.460 | -2.247 |
| bta-miR-346 | TGTCTGCCCGCATGCCTGCCTCT | hsa-miR-346 | -5.105 | -4.126 | -2.229 |
| bta-miR-362-3p | AACACACCTATTCAAGGATTC | hsa-miR-362-3p | -4.321 | -3.358 | 0.670 |
| bta-miR-362-5p | AATCCTTGGAACCTAGGTGTGAGT | hsa-miR-362-5p | -0.050 | -0.740 | -1.662 |
| bta-miR-363 | ATTGCACGGTATCCATCTGCG | hsa-miR-363-5p | 2.492 | 2.755 | 3.406 |
| bta-miR-365-5p | AGGGACTTTTGGGGGCAGATGTG | hsa-miR-365a-3p | -2.853 | -2.429 | -0.204 |
| bta-miR-369-3p | AATAATACATGGTTGATCTTT | hsa-miR-369-3p | -3.420 | -0.357 | -0.898 |
| bta-miR-374a | TTATAATACAACCTGATAAGTG | hsa-miR-374a-5p | 1.729 | 1.992 | 2.009 |
| bta-miR-374b | ATATAATACAACCTGCTAAGTG | hsa-miR-374b-5p | 4.017 | 3.395 | 2.697 |
| bta-miR-375 | TTTTGTTCGTTCGGCTCGCGTGA | hsa-miR-375 | -3.216 | -1.016 | 0.006 |
| bta-miR-380-3p | TATGTAATGTGGTCCACGTCT | hsa-miR-380-5p | -0.014 | 1.893 | 1.578 |
| bta-miR-381 | TATACAAGGGCAAGCTCTCTGT | hsa-miR-381-3p | -3.471 | -1.460 | -2.247 |
| bta-miR-382 | GAAGTTGTTCGTGGTGGATTCG | hsa-miR-382-5p | 0.368 | 0.227 | 2.237 |
| bta-miR-433 | ATCATGATGGGCTCCTCGGTGT | hsa-miR-433-3p | -5.832 | -3.703 | -0.766 |
| bta-miR-451 | AAACCGTTACCATTACTGAGTTT | hsa-miR-451a | 2.357 | 0.382 | 0.632 |
| bta-miR-452 | TGTTTGCAGAGGAAACTGAGAC | hsa-miR-452-5p | -3.850 | -1.781 | -2.646 |
| bta-miR-454 | TAGTGCAATATTGCTTATAGGGT | hsa-miR-454-3p | -0.039 | -2.026 | 1.159 |
| bta-miR-483 | TCACTCCTCTCCTCCCGTCTT | hsa-miR-483-3p | -6.157 | -1.976 | -0.461 |
| bta-miR-485 | AGAGGCTGGCCGTGATGAATTCG | hsa-miR-485-3p | -4.208 | -2.062 | -0.635 |
| bta-miR-487b | AATCGTACAGGGTCATCCACTT | hsa-miR-487b-3p | -1.724 | -0.581 | -0.322 |
| bta-miR-494 | TGAAACATACACGGGAAACCTC | hsa-miR-494-3p | 0.428 | 1.792 | 0.996 |
| bta-miR-495 | AAACAAACATGGTGCACTTCTT | hsa-miR-495-3p | -3.775 | -1.665 | -2.615 |
| bta-miR-499 | TTAAGACTTGCAGTGATGTTT | hsa-miR-499a-5p | -2.219 | -3.406 | 0.163 |
| bta-miR-502a | AATGCACCTGGGCAAGGATTCA | hsa-miR-502-3p | -1.722 | -1.948 | -2.182 |
| bta-miR-543 | AAACATTCGCGGTGCACTTCTT | hsa-miR-543 | -2.303 | -1.404 | -0.568 |
| bta-miR-655 | ATAATACATGGTTAACCTCTCT | hsa-miR-655-5p | -2.543 | -3.581 | -3.406 |
| bta-miR-671 | AGGAAGCCCTGGAGGGGCTGGAG | hsa-miR-671-3p | -2.250 | -2.875 | -0.054 |
| bta-miR-874 | CTGCCCTGGCCCGAGGGACCGA | hsa-miR-874-3p | -1.700 | 0.122 | -0.244 |
| bta-miR-885 | TCCATTACACTACCCTGCCTCT | hsa-miR-885-5p | -3.679 | -1.452 | 0.571 |
| bta-miR-92b | TATTGCACTCGTCCCGGCCTCC | hsa-miR-92b-3p | -3.611 | -1.626 | -0.262 |
| bta-miR-98 | TGAGGTAGTAAGTTGTATTGTT | hsa-miR-98-5p | 1.772 | -0.439 | -0.442 |
